# CD44+ and CD31+ extracellular vesicles (EVs) are significantly reduced in polytraumatized patients with hemorrhagic shock – evaluation of their diagnostic and prognostic potential

**DOI:** 10.3389/fimmu.2023.1196241

**Published:** 2023-08-18

**Authors:** Birte Weber, Ramona Sturm, Dirk Henrich, Ingo Marzi, Liudmila Leppik

**Affiliations:** Department of Trauma−, Hand− and Reconstructive Surgery, University Hospital Frankfurt, Goethe-University, Frankfurt am Main, Germany

**Keywords:** polytrauma, extracellular vesicles, exosomes, size exclusion chromatography, hemorrhagic shock, blood transfusion, MACSPlex

## Abstract

**Background:**

Hemorrhagic shock (HS) is responsible for approximately 2 million deaths per year worldwide and is caused in 80% by polytrauma. These patients need a precise and quick diagnostic, which should be based on a combination of laboratory markers and radiological data. Extracellular vesicles (EVs) were described as potential new markers and mediators in trauma. The aim of the present study was to analyze, whether the surface epitopes of plasma-EVs reflect HS in polytraumatized patients and whether cell-specific EV subpopulations are useful diagnostic tools.

**Material and methods:**

Plasma samples from polytraumatized patients (ISS ≥16) with HS (n=10) and without (n=15), were collected at emergency room (ER) and 24h after trauma. Plasma-EVs were isolated via size exclusion chromatography and EV-concentrations were detected by Coomassie Plus (Bradford) Assay. The EVs subpopulations were investigated by a bead-based multiplex flow cytometry measurement of surface epitopes and were compared with healthy controls (n=10). To investigate the diagnostic and prognostic potential of EVs subpopulations, results were correlated with clinical outcome parameters documented in the electronical patients’ record.

**Results:**

We observed a significant reduction of the total amount of plasma EVs in polytrauma patients with HS, as compared to polytrauma patients without HS and healthy controls. We found significant reduction of CD42a+ and CD41b+ (platelet-derived) EVs in all polytrauma patients, as well as a reduction of CD29+ EVs compared to healthy volunteers (*p<0.05). CD44+ and CD31+ EVs were specifically altered in patients with HS (*p<0.05). Both EV populations showed a moderate correlation (r² = 0.42) with the transfusion of erythrocyte concentrate, were associated with non-survival and the need for catecholamines (*p<0.05).

**Conclusion:**

Our data reveal that polytrauma patients with a hemorrhagic shock are characterized by a reduction of CD44+ and CD31+ plasma-EVs. Both EV populations showed a moderate correlation with the need of erythrocyte transfusion, were associated with non-survival and the need for catecholamines.

## 1 Introduction

Trauma continues to be one of the world’s top causes of morbidity and mortality (1). Hemorrhagic shock (HS) is responsible for approximately 2 million deaths per year worldwide and in 80% is caused by polytrauma (2). Polytraumatic injury has complex nature and includes simultaneous injury of multiple body regions and organs and severe wounds with a significant loss of soft tissue, which all lead to hemorrhage and shock, extended systemic inflammation, multi-organ failure and as a result – to death (3–5). In polytraumatized patient’s exsanguination is reported as the primary preventable cause of death in the first 24h after injury (6–8). Often severe injured patients need massive or large volume transfusion (≥ 10 units of packed red blood cells in a 24-hour period) of blood/blood products (9, 10). In patients receiving massive volume transfusion, the severity of injury and subsequent profound blood loss lead to up to 15% higher mortality risk at 24 hours (11). The required care for polytraumatized patients, as well as the large amount of transfusion products need a large number of well-trained clinical staff and expensive medical equipment, that result in a high economic burden for the society (12). A secondary consequence of hemorrhagic shock is traumatic hypovolemia, which triggers a rapid and substantial loss of blood, tissue injury and hypoxia. They cause secondary injuries including coagulopathy, endotheliopathy, microcirculation failure, inflammation, and immune activation. Consequently, these dysfunctions lead to secondary organ failures and multi-organ failure (13, 14). All in all, these patients need a precise and quick diagnostic, which should be based on a combination of laboratory markers and radiological data.

The extracellular vesicles (EVs) may be a new and useful laboratory marker for the identification of trauma severity and complications (15). EVs are small, lipid-bilayer covered particles released by cells, which lacks a functional nucleus (16). These particles are known to participate in cell-cell communications and transport biologically active components and mediators such as RNAs, DNAs, and proteins in their cargo. EV surface epitopes were also shown to reflect their cellular origin, which could provide an insight into cell communication mechanisms and therefore help to understand the cellular interplay after trauma (17).

Only a few studies analyze EVs or exosomes after hemorrhagic shock. One working-group described in 2018 that alveolar macrophages after hemorrhagic shock released exosomes, which induced the production of reactive oxygen species in polymorphonuclear neutrophils (PMNs) and therefore led to necroptosis (18). In the present literature, the study analyzing release of EV after HS and the cellular origin of these EVs are still lacking. In order to address this lack, we performed a comparative analysis of surface epitopes/cellular origin of EVs circulating in plasma of polytraumatized patients with and without HS and healthy controls by using a multiplex bead-based platform. Obtained EVs surface antigen signature was then correlated with the patient’s clinical outcome parameters and the need for transfusion.

## 2 Material and methods

### 2.1 Study design

The present study was ethically approved by the Local Ethics Committee of the University of Frankfurt (approval ID 89/19). In the present study plasma-EVs from multiple injured patients (polytrauma, PT) (ISS≥16, n = 16) were compared with plasma EVs from healthy volunteers (n=10), as well as patients with an initial hemorrhagic shock (HS) (n = 10). Inclusion criteria were: clinical diagnosis of shock due a positive shock index in the emergency room, initial hemoglobin ≤ 10 mg/dl, lactate ≥20 mg/dl, need for catecholamines, receive of ≥10 erythrocyte concentration in the first 24h). Patients with trauma who were hospitalized to a Level 1 Trauma Center between 2016 and 2020 were included in this study. Blood collections time points were: admission to the emergency room (ER) and 24 hours later. Collected blood samples were centrifuged for 15 minutes at 3500g and 4°C to isolate plasma. Plasma samples from healthy participants were handled in the same manner as samples from patients. Based on the patient’s electronical record, outcome parameters (time in the hospital, ventilation time, time on ICU/IMC, blood transfusion, need for catecholamines etc.) were documented and correlated with the results from the MACSPlex measurements.

### 2.2 EV isolation and characterization

Extracellular vesicles were isolated from 100µl plasma by size exclusion chromatography (Exo-Spin mini, Cell guidance systems, Cambridge, UK) as described by manufacturer. EV protein concentration was measured by Coomassie Plus (Bradford) Asssay (Thermo Fisher Scientific, Rockford, IL, USA).

### 2.3 Western blot

For CD63 and CD9 proteins expression analysis, 4µg EV protein from each sample were separated by electrophoresis in 10% sodium dodecyl polyacrylamide gel (SDS-PAGE). Afterwards, the transfer of proteins to the polyvinylidene fluoride membrane (BioRad Laboratories, Hercules, CA, USA) was conducted. The blocking of the membrane was performed overnight in blocking solution (5% milk in TBST buffer) at +4°C. Afterwards, the membrane was incubated for 1h at room temperature with primary antibody against CD63 (Invitrogen, 10628D, 1:1000) or CD9 (Invitrogen, 10626D, 1:1000). After several washes, membrane was incubated with conjugated secondary antibody (horseradish peroxidase-linked, 1:2000, Cell signalling Technology, Leiden, The Netherlands) in blocking buffer at room temperature for 1h. For signal development, ECL™ Western-Blotting-Reagent (MERCK, RPN2016, Taufkirchen, Germany) was applied according to the manufacturer’ instruction.

### 2.4 EVs’ surface epitopes profiling

Plasma EV samples were subjected to surface epitopes bead-based flow cytometry analysis (MACSPlex Exosome kit Miltenyi Biotec, Bergisch Gladbach, Germany) according to the manufacturer protocol. In brief, 20µg EVs from each of polytrauma- (n = 16) or hemorrhagic shock- (n= 10) patients’ and healthy controls’ (n= 10) samples were diluted in MACSPlex buffer to a total volume of 120 µl and incubated with MACSPlex exosome capture beads overnight. APC-labeled CD6, CD63 and CD81 antibodies were used for counterstaining. APC-A values were acquired with BD FACSCanto II cytometer (BD biosciences, Heidelberg, Germany) and FACS DIVA software (BD biosciences, Heidelberg, Germany) was used to analyze the data. For each sample the median signal intensity of the signals detected for the CD9, CD63 and CD81 capture beads were calculated and their geometrical mean was used as the normalization factor for each sample.

### 2.5 Statistical analysis

The data are presented as mean ± standard error of the mean (SEM). Kolmogoroff-Smirnow-Lilleforts-Test was used to proof the normal distribution of the data. The difference among the groups was validated with one-way ANOVA followed by Dunnettes/Turkeys multiple comparison test. In case of two groups comparison, T-test was applied. A linear dependence between two variables was measured as Pearson correlation (r). Graph Pad-Prism 9 was used for all statistical analysis. A p -value of 0.05 or less is regarded as statistically significant.

## 3 Results

The present study was conducted in 26 polytraumatized patients with an ISS of ≥16 (16 polytraumatized patients without and 10 patients with hemorrhagic shock) and 10 healthy volunteers. The isolated EVs were shown to express exosome-specific tetraspanins CD9 and CD63 (shown by Western blot).

**Figure 1:**
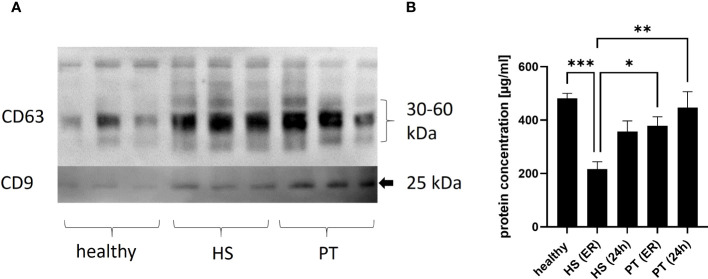
Concentration of plasma EVs. **(A)** Representative image of EV Western blot, isolated from healthy plasma vs. polytrauma (PT) plasma vs hemorrhagic shock. 1 **(B)** Results of plasma EVs’ quantification analysis in healthy controls and polytrauma patients with (HS) or without (PT) hemorrhagic shock. ER - in the emergency room, 24h -24h after admission in the hospital. *p = ≤0.05; **p ≤ 0.01, ***p ≤ 0.001.

The plasma concentration of EVs (measured by protein concentration) was found to differ significantly between the investigated groups. In details, the plasma EV concentration measured in hemorrhagic shock patients in the ER was significantly lower as plasma EV concentration in healthy volunteers and polytrauma patients (without hemorrhagic shock, ER and 24 hrs) (**Figure 1B**).

**Figure 2:**
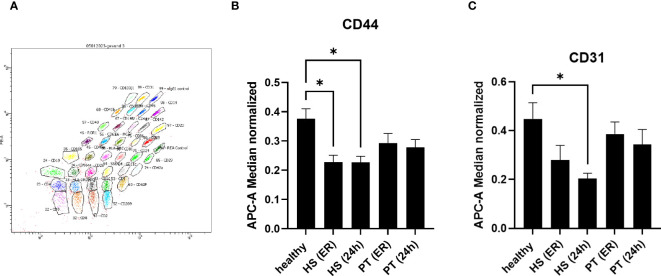
MACSPlex analysis of EVs surface epitopes – specific changes in hemorrhagic shock patients. 2 **(A)** Representative image of MACSPlex EVs flow cytometry analysis, healthy volunteers EVs sample. 2 **(B)** Amount of CD44+ EVs was significantly reduced in the plasma of hemorrhagic shock patients (HS) as compared to healthy volunteers’ group. 2 **(C)** Amount of CD31+ EVs was significantly reduced in hemorrhagic shock patients at 24h after admission as compared to the healthy controls. PT, polytrauma; ER, emergency room, *p ≤0.05.

The presence of specific surface epitopes in plasma EVs was investigated with multiplex flow cytometry MACSPlex analysis (**Figure 2A**; **Table 1**). Our findings demonstrated that across the 37 examined epitopes, CD44 was significantly (p ≤ 0.05) decreased in the hemorrhagic shock samples at both time points, as compared to the healthy controls’ samples (**Figure 2B**). CD31 was also significantly reduced in this group of patients, as compared to healthy controls, but only at 24h time point (**Figure 2C**).

**Table 1:** Expression of EVs surface epitopes in patients and healthy controls, MACSPlex measurements.

	Healthy (Mean ± SD)	HS (Mean ± SD) ER	HS (Mean ± SD) 24h	PT (Mean ± SD) ER	PT (Mean ± SD) 24h
CD19	0.14 ± 0.08	0.15 ± 0.07	0.10 ± 0.03	0.16 ± 0.09	0.16 ± 0.06
CD4	0.16 ± 0.10	0,21 ± 0.22	0.11 ± 0.05	0.17 ± 0.09	0.17 ± 0.08
CD3	0.19 ± 0.07	0.23 ± 0.18	0.14 ± 0.08	0.20 ± 0.11	0.18 ± 0.08
CD105	0.27 ± 0.12	0.30 ± 0.12	0.25 ± 0.10	0.30 ± 0.10	0.28 ± 0.11
CD56	0.24 ± 0.08	0.18 ± 0.11	0.15 ± 0.06	0.25 ± 0.12	0.16 ± 0.08
HLA-DRDPDQ	0.35 ± 0.08	0.40 ± 0.15	0.33 ± 0.09	0.33 ± 0.14	0.28 ± 0.09
CD8	0.39 ± 0.16	0.38 ± 0.18	0.32 ± 0.11	0.44 ± 0.24	0.40 ± 0.17
ROR1	0.39 ± 0.32	0.21 ± 0.08	0.27 ± 0.05	0.25 ± 0.16	0.29 ± 0.14
CD49e	0.35 ± 0.34	0.26 ± 0.18	0.17 ± 0.07	0.34 ± 0.23	0.27 ± 0.12
CD25	0.22 ± 0.14	0.26 ± 0.19	0.18 ± 0.07	0.24 ± 0.18	0.21 ± 0.07
CD1c	0.15 ± 0.07	0.25 ± 0.28	0.10 ± 0.07	0.17 ± 0.08	0.15 ± 0.07
CD2	0.20 ± 0.11	0.23 ± 0.19	0.12 ± 0.05	0.21 ± 0.19	0.16 ± 0.07
CD40	0.41 ± 0.28	0.21 ± 0.12	0.17 ± 0.05	0.35 ± 0.29	0.32 ± 0.16
HLA-ABC	0.21 ± 0.09	0.17 ± 0.11	0.16 ± 0.08	0.26 ± 0.22	0.24 ± 0.09
SSEA-4	0.23 ± 0.13	0.20 ± 0.15	0.18 ± 0.07	0.24 ± 0.12	0.21 ± 0.08
CD209	0.19 ± 0.08	0.19 ± 0.20	0.12 ± 0.04	0.20 ± 0.11	0.16 ± 0.07
CD41b	1.61 ± 1.04	0.73 ± 0.29*	0.63 ± 0.23**	1.02 ± 0.49	0.75 ± 0.46*
CD146	0.26 ± 0.12	0.25 ± 0.12	0.20 ± 0.06	0.29 ± 0.16	0.25 ± 0.15
MCSP	0.17 ± 0.10	0.15 ± 0.10	0.14 ± 0.04	0.19 ± 0.13	0.20 ± 0.07
CD11c	0.16 ± 0.10	0.14 ± 0.09	0.15 ± 0.07	0.18 ± 0.13	0.18 ± 0.10
CD62P	0.64 ± 0.45	0.77 ± 0.45	0.27 ± 0.16	0.82 ± 0.52	0.26 ± 0.11
CD133/1	0.32 ± 0.23	0.20 ± 0.04	0.23 ± 0.05	0.28 ± 0.16	0.26 ± 0.10
CD326	0.35 ± 0.25	0.27 ± 0.15	0.28 ± 0.07	0.29 ± 0.14	0.26 ± 0.09
CD44	0.38 ± 0.10	0.23 ± 0.06*	0.23 ± 0.06*	0.29 ± 0.12	0.28 ± 0.09
CD86	0.18 ± 0.13	0.18 ± 0.14	0.14 ± 0.05	0.22 ± 0.15	0.18 ± 0.07
CD24	0.27 ± 0.15	0.21 ± 0.15	0.22 ± 0.07	0.23 ± 0.12	0.26 ± 0.09
CD42a	4.44 ± 4.79	0.45 ± 0.11**	0.73 ± 0.71**	0.63 ± 0.37**	0.60 ± 0.39**
CD31	0.45 ± 0.20	0.28 ± 0.18	0.20 ± 0.06*	0.39 ± 0.19	0.34 ± 0.22
CD45	0.21 ± 0.07	0.31 ± 0.23	0.17 ± 0.08	0.24 ± 0.18	0.19 ± 0.09
CD142	0.20 ± 0.10	0.14 ± 0.08	0.14 ± 0.04	0.24 ± 0.13	0.20 ± 0.08
CD69	0.32 ± 0.26	0.23 ± 0.13	0.21 ± 0.06	0.28 ± 0.14	0.28 ± 0.12
CD29	1.40 ± 1.00	0.71 ± 0.29*	0.62 ± 0.18*	0.75 ± 0.23*	0.70 ± 0.29*
CD14	0.29 ± 0.15	0.30 ± 0.24	0.19 ± 0.06	0.24 ± 0.17	0.23 ± 0.08
CD20	0.18 ± 0.11	0.15 ± 0.07	0.15 ± 0.06	0.20 ± 0.14	0.20 ± 0.11

HS, hemorrhagic shock; PT, polytrauma; ER, emergency room; *p = ≤0.05; **p ≤ 0.01 compared to healthy controls.

**Figure 3:**
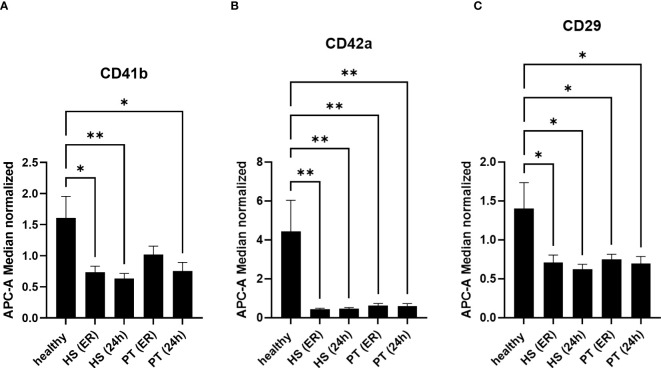
EVs-surface epitopes expression in groups of polytraumatized patients with and without hemorrhagic shock. 3 **(A)** The number of CD41b+ EVs is significantly reduced in polytrauma patients’ groups with and without hemorrhagic shock. 3 **(B)** Amount of CD42a+ EVs is significantly reduced in all groups of patients at all investigated timepoints. 3 **(C)** CD29+ EVs are significantly reduced in all patients compared to healthy controls. *p ≤ 0.05 and **p ≤ 0.01. PT, polytrauma; HS, hemorrhagic shock; ER, emergency room.

Next to the specific changes in the hemorrhagic shock group, further changes in EVs populations in polytrauma patients as compared to healthy controls were observed (**Figure 3**). Out of 37 analyzed epitopes, 3 have shown differential expression in both polytrauma patients’ groups. As compared to the healthy controls, CD41b^+^ EVs were found to be significantly reduced in the polytrauma group after 24h, as well as at both time points in the hemorrhagic shock patients (**Figure 3A**). CD42a^+^ EVs were reduced in all groups of patients in comparison to healthy controls (**Figure 3B**). Furthermore, the amount of CD29^+^ EVs was also reduced in all groups of patients in comparison to healthy volunteers (**Figure 3C**).

**Figure 4:**
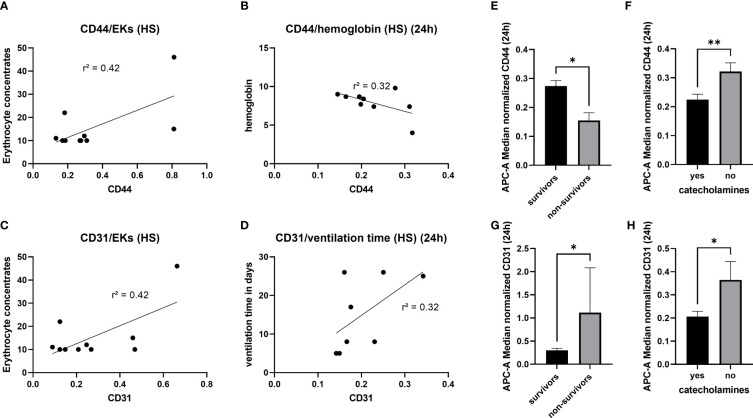
Diagnostic and prognostic potential of CD44+- and CD31+- EVs. 4 **(A)** Moderate correlation between the amount of CD44+ EVs and the erythrocyte concentrates transfused in the first 24h. 4 **(B)** Negative correlation between the CD44+ EVs and the haemoglobin level, measured 24h after hemorrhagic shock. Association between CD31+ EVs and the amount of transfused erythrocyte concentrates 4 **(C)**, as well as the ventilation time 4 **(D)**. Significantly reduced amount of CD44+ EVs in non-survivors 4 **(E)** and patients with need of catecholamines 4 **(F)**. Increased amount of CD31+ EVs in non-survivors **(G)** and reduction of CD31+ EVs in patients with need of catecholamines **(H)**. *p ≤ 0.05 and **p ≤ 0.01. PT, polytrauma; HS, hemorrhagic shock; ER, emergency room; EK, erythrocyte concentrate.

In order to analyze the diagnostic and prognostic potential of the amount of CD44^+^ EVs und CD31^+^EVs, correlation analysis with clinical outcome parameters (**Table 2**) as well as the amount of blood transfusions was conducted. In hemorrhagic shock patients, amount CD44^+^ and CD31^+^ EVs correlated moderate (r^2 = ^0.42) with the amount of erythrocyte concentrates transfused in the first 24h (**Figure 4A, C**). No correlation between the CD44+ or CD31+ exosomes and the amount of platelet/fresh frozen plasma transfusion was observed (**Supplementary Figure S3**). Also, moderate negative correlation (r^2 = ^0.32) was found among the CD44^+^ EVs and the hemoglobin level in patients with hemorrhagic shock at 24h time point (**Figure 4B**). After 24h, the amount of CD31^+^ EVs correlated moderate (r^2 = ^0.32) with the ventilation time (**Figure 4D**). In all the polytrauma patients, the amount of CD44^+^ EVs was significantly reduced in non-survivors (**Figure 4E**), while amount of CD31+ EVs was significantly increased in non-survivors (**Figure 4G**). In all polytraumatized patients, the need of catecholamines was associated with significantly decreased CD44^+^- and CD31^+^- EV levels (**Figure 4F, H**). In addition, weak correlation was found among expression of these epitopes and hematocrit in HS group of patients (**Supplementary Table S1**), but not with lactate and crystalloids fluidics.

**Table 2:** Patients’ clinical data.

	HS group	PT group
Mean age [years] ± SD	45 ± 24.6	55.7 ± 16.7
Male [%]	70	87.5
ISS ± SD	53.8 ± 14.6	26.6 ± 9.1****
Initial hemoglobin [g/dl] ± SD	8.1 ± 1.7	14 ± 0.6****
Hemoglobin (24h) [g/dl] ± SD	8.3 ± 0.8	11.9 ± 1.73****
Initial hematocrit [%] ± SD	27 ± 7.8	42 ± 2.3 ***
Hematocrit (24h) [%] ± SD	22 ± 4.5	35 ± 5.7****
Initial lactate [mg/dl] ± SD	57.7 ± 22.8	13.7 ± 3.93****
Lactate (24h) [mg/dl] ± SD	46 ± 24.9	15 ± 13.3****
Initial platelet count [/µl] ± SD	145 ± 77.5	217.1 ± 37.3**
Platelet count (24h) [/µl] ± SD	134 ± 36.9	172.9 ± 30.6*
Initial quick [%] ± SD	58 ± 22.9	92.9 ± 15.0***
Quick (24h) [%] ± SD	71.1 ± 17.5	82.2 ± 13.5
Initial INR ± SD	1.87 ± 1.3	1.1 ± 0.13***
INR (24h) ± SD	1.31 ± 0.2	1.2 ± 0.13
Initial aPTT [sec] ± SD	36.0 ± 8.5	24.4 ± 2.18****
aPTT (24h) [sec] ± SD	31 ± 5.2	25.1 ± 1.73**
Time on ICU/IMC [days] ± SD	18.9 ± 12.3	9.6 ± 6.8*
Ventilation time[days] ± SD	13.6 ± 9.4	4.1 ± 6.4**
Non-survivors [%]	30	6.25
Need for catecholamines [%]	100	37.5
Erythrocyte concentrates (in the first 24h [number a 330 ml] ± SD	16 ± 10.8	0.2 ± 0.73****
Platelet concentrates (in the first 24h) [number a 250 ml] ± SD	4.2 ± 3.3	0****
Tranexamic acid (in the first 24h) [number a 1g] ± SD	1.3 ± 0.5	0.1 ± 0.24****
Fresh Frozen Plasma (in the first 24h) [number a 250 ml] ± SD	10 ± 9.3	0****
PBSB (in the first 24h) [IE] ± SD	2277.8 ± 1271.7	0
Crystalloids (in the first 24h) [ml] ± SD	6333 ± 1986	3167 ± 1929**

INR, International Normalized Ratio; aPTT, activated partial thromboplastin time; ICU/IMC, Intensive Care Unit/Intermediate Care; PBSB, Prothrombin complex concentrate; *p = ≤0.05; **p ≤ 0.01, ***p ≤ 0.001, ****p ≤ 0.0001.

## 4 Discussion

In the present study we hypothesized, that severe conditions as hemorrhagic shock not only dramatically influence cellular activity but also affect EVs production and that such changes should be reflected in the plasma-EV repertoire. If true, then EVs, floating in the plasma of polytrauma patients with and without hemorrhagic shock differs in their cellular origin and therefore could be used as diagnostic tools to detect a life-threatening hemorrhage. To proof this hypothesis, we isolated EVs and compared the patterns of plasma EVs-surface epitopes in polytrauma patients with and without hemorrhagic shock, as well as healthy controls.

First of all, the amount of isolated EVs from patients with hemorrhagic shock (ER) was significantly lower compared to healthy controls and the polytrauma patients’ group (**Figure 1**). These results showed that patients with hemorrhagic shock should be seen as separate group of polytrauma patients, as EVs counts typically is increased in trauma patients as compared to healthy controls (17, 19, 20). Reduction of EVs in HS might be explained by reduction (due to consumption) of some EV-releasing types of cells. For example, consumption of platelets due to hemorrhage could lead to decrease of platelet derived (CD42a+/CD41b+) EVs. In the present study, we observed in both polytrauma groups (with and without hemorrhagic shock) significant reduction of CD42a+ EVs (**Figure 3**). However, we were not able to show correlation between amount of CD42+ EVs and the total amount of platelets, as well as the need for transfusion of platelet concentrates (data not shown), in these patients. The CD41b+EV population was also reduced in all trauma groups. In a few previous study platelet-derived microparticles were found to be increased in polytrauma patients (21, 22), that contradict our findings. However, other studies rather focused on other size populations of extracellular vesicles and therefore might be not comparable with the present results. The role of platelet-derived EVs in polytrauma was also investigated in some animal models. For example, EVs were found to decrease endothelial cell permeability and to restore endothelial cell junctions in a tail snip hemorrhagic shock model in vivo (23). Furthermore, in a rat model of traumatic hemorrhagic shock, platelet-EVs were found to maintain hemodynamic stability and to attenuate uncontrolled bleeding (23). These in vivo findings together with our patients’ data show the importance of platelet derived EVs in the hemorrhagic shock outcomes and the need for further studies.

In addition, we showed that the amount of CD29+ EVs is significantly reduced in both polytrauma groups. CD29 is widely expressed by different types of cells, including platelets, cytotoxic T-cells, endothelial-, stem- and cancer- cells, therefore CD29+ EVs could have mixed cell origin. ITGB1 (Integrin Subunit Beta 1 = CD29) is a 110 kDa cell surface glycoprotein that is widely expressed by a variety of cells including all leucocytes. ITGB1 forms non-covalently linked heterodimers with at least six different alpha chains determining the binding properties of beta1 integrins (24). Next to other roles, integrins mediate leukocyte migration and activation triggered by inflammatory mediators. Integrin is indispensable for maintaining productive leukocyte migration. Therefore, a reduction of CD29-enriched EVs also might influence the migration and activation of leucocytes after trauma and hemorrhage (25). Also, CD29-encriched EVs were described to mediate monocyte adhesion and promote liver inflammation in a murine model of non-alcoholic steatohepatitis (26). As cell adhesion receptor, ITGB1 was also described to influence the tissue factor release on procoagulant EVs dismissed by endothelial cells (27). In hemorrhagic shock, further studies will be needed to clarify the function and origin of these CD29+EVs.

A crucial role of EVs in trauma and hemorrhage has been demonstrated by the fact that HS-derived extracellular vesicles can induce an endothelial dysfunction and coagulopathy when transferred in heathy animals (28). Unfortunately, this study did not analyze which EV-populations were released after traumatic HS and therefore are responsible for the unfavorable outcome. In the present study, as one of the main results, we found significant reduction of the CD44+ and the CD31+ EVs in the HS-patients. CD44 is a transmembrane glycoprotein acting as cell surface adhesion receptor. The ubiquitous transmembrane cell surface molecule CD44 is widely distributed in normal adult tissue, but is also highly expressed in many tumour cells (29). Variant CD44 isoforms are expressed on hematopoietic cells, in keratinocytes, macrophages, epithelial cells, but also in the central nervous system (29). In contrast to the research studies focused on the role of CD44+EVs in cancer, studies on the role of these EVs in trauma and/or hemorrhage are very rare. A CD44 knockout - study in myocardial infarction model showed the important role of CD44+ EVs in angiogenesis (30). Furthermore, CD44+ EVs are described to affect the leukocyte proliferation (31). Earlier studies connecting CD44+EVs and hemorrhagic shock, were missing in the literature. In the present study, the reduction of CD44+ EVs in hemorrhagic shock patients was associated with non-survival, need of catecholamines and the CD44+ EV concentration was correlated moderately with the need for erythrocyte transfusion (**Figure 4**). These suggest that the subpopulation of CD44+ EVs might be a useful diagnostic and prognostic marker in hemorrhagic shock patients.

Next to CD44+ EVs, we found the moderate correlation between the need for erythrocyte concentrates and amount of CD31+ EVs and showed that reduction of CD31+ EVs is connected to the need of catecholamines (**Figure 4**). CD31 (Platelet endothelial cell adhesion molecule/PECAM-1)+ EVs could be released by monocytes, platelets, granulocytes, endothelial cells, lymphocytes and epithelial cells (32). CD31 is a cell-cell adhesion protein, which is highly expressed at endothelial cell-cell junctions. It is working as a stress-response protein involved in the maintenance of endothelial cell junctional integrity and restoration of vascular permeability barrier following inflammatory or thrombotic challenges (32). CD31+ EVs were described in high blood pressure patients (33). One could speculate, that a loss/reduction of CD31+ EVs might be associated with a loss of cell junction integrity and consequently with decreased restoration of vascular permeability due inflammatory damage. The role of CD31+ EVs in endothelial integrity has not been investigated before.

The findings of this study being novel and therefore, we believe, important for the polytrauma and hemorrhagic shock research, should be interpreted with caution due to several study limitations. First, the group of patients with polytrauma and HS is characterized by significantly higher ISS as compared to the polytrauma patients without initial hemorrhagic shock. To investigate the influence of the ISS on the results of the present study, we conducted correlation analysis and found that ISS did not correlate with the amount of CD44+ (r² = 0.02) or CD31+ (r² = 0.04) EVs. Next to the ISS, also, the low number of patients in this group might be a limitation of the study, but unfortunately, it was not possible to enroll more patients fulfilling the study criteria. Furthermore, as the hemorrhagic shock patients normally receive significant amounts of crystalloid fluidics, their plasma samples might be diluted as compared to other patients or controls. This effect is not expected to be major, as EV-epitopes expression study involves the use of the same amount of EV proteins and normalization of data to exosomal marker expression (CD9/63/81) for each sample, but it could not be completely excluded. The observed weak correlation of hematocrit with the expression of CD31 and CD44 EV-epitopes in HS group supports this awareness and future studies should be performed with the detailed control of given amounts of fluidics and blood products in order to clarify this issue. In addition, the exosome isolation method used in this study (SEC) might influence the results. Nowadays, there is no gold standard method for EV isolation as each of the methods has its own limitations. We used SEC, as it provides relevant quantity and quality of EV isolates, is reproducible, scalable and applicable for the low-volume samples, however the possible contamination of EV-isolates with plasma proteins should be considered.

Overall, the results of this study performed with two polytrauma patients’ groups (with and without HS) demonstrate that HS has effect on the number and repertoire of plasma EVs. Some EVs populations (CD44+ and CD31+) were found to correlate with the need for catecholamines, blood transfusion and the survival. CD44+ and CD31+ EVs should be investigated in future studies as potential diagnostic tool of HS.

## 5 Conclusion

Our comparative analysis of plasma EVs’ populations with different cellular origin/epitope expression revealed reduction of CD41b+, CD42a+ and CD29+ EVs in all polytrauma patients regardless of hemorrhagic shock. At the same time, reduction of CD44+ and CD31+ plasma-EVs populations was found specific for the polytrauma patients with hemorrhagic shock. Both these EV populations showed a moderate correlation with the erythrocyte transfusion require and were associated with the need for catecholamines and non-survival.

## Data availability statement

The original contributions presented in the study are included in the article/**Supplementary Material**. Further inquiries can be directed to the corresponding author.

## Ethics statement

The studies involving human participants were reviewed and approved by the Local Ethics Committee of the University of Frankfurt (approval ID 89/19). The patients/participants provided their written informed consent to participate in this study.

## Author contributions

BW, DH, RS, LL and IM substantial contributed to the conception or design of the work. BW, LL, DH, RS are responsible for the analysis and the interpretation of data for the work. BW, DH, LL, IM drafting the work or revising it critically for important intellectual content. All authors provide approval for publication of the content and agree to be accountable for all aspects of the work in ensuring that questions related to the accuracy or integrity of any part of the work are appropriately investigated and resolved. All authors contributed to the article and approved the submitted version.

## Supplementary material

Supplementary Figure 1 Representative results of nanoparticle tracking analysis (NTA) of extracellular vesicles, isolated from patients’ plasma.

Supplementary Figure 2 Representative images of Westernblot analysis of CD9 (left) and CD63 (right).

Supplementary Figure 3 Correlation Analysis of Fresh Frozen Plasma (FFPs) and platelet concentrates (TKs) and CD31/CD44+ EVs. All = both polytrauma groups, with and without hemorrhagic shock, HS = hemorrhagic shock

Supplementary Table 1 Correlation analysis of EV epitopes and lactate, hematocrit and crystalloids fluidics in HS group and in all patients.
