# Supplementary figures and images for: CD44+ and CD31+ extracellular vesicles (EVs) are significantly reduced in polytraumatized patients with hemorrhagic shock – evaluation of their diagnostic and prognostic potential
Source: Front Immunol. 2023 Aug 18;14:1196241. doi: 10.3389/fimmu.2023.1196241 (PMC10471799; doi:10.3389/fimmu.2023.1196241)

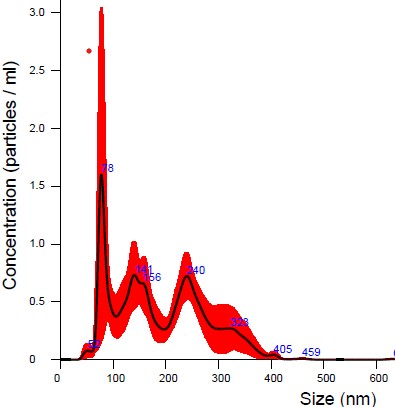

Supplement: Supplementary Figure 1 — Representative results of nanoparticle tracking analysis (NTA) of extracellular vesicles, isolated from patients’ plasma. [file Image_1.jpeg]

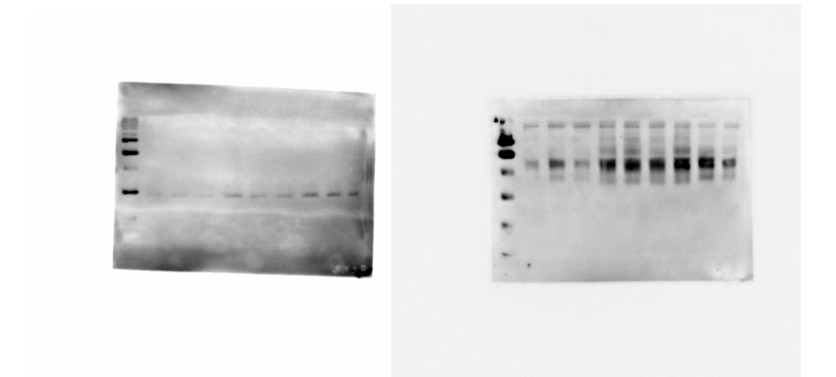

Supplement: Supplementary Figure 2 — Representative images of Westernblot analysis of CD9 (left) and CD63 (right). [file Image_2.jpeg]

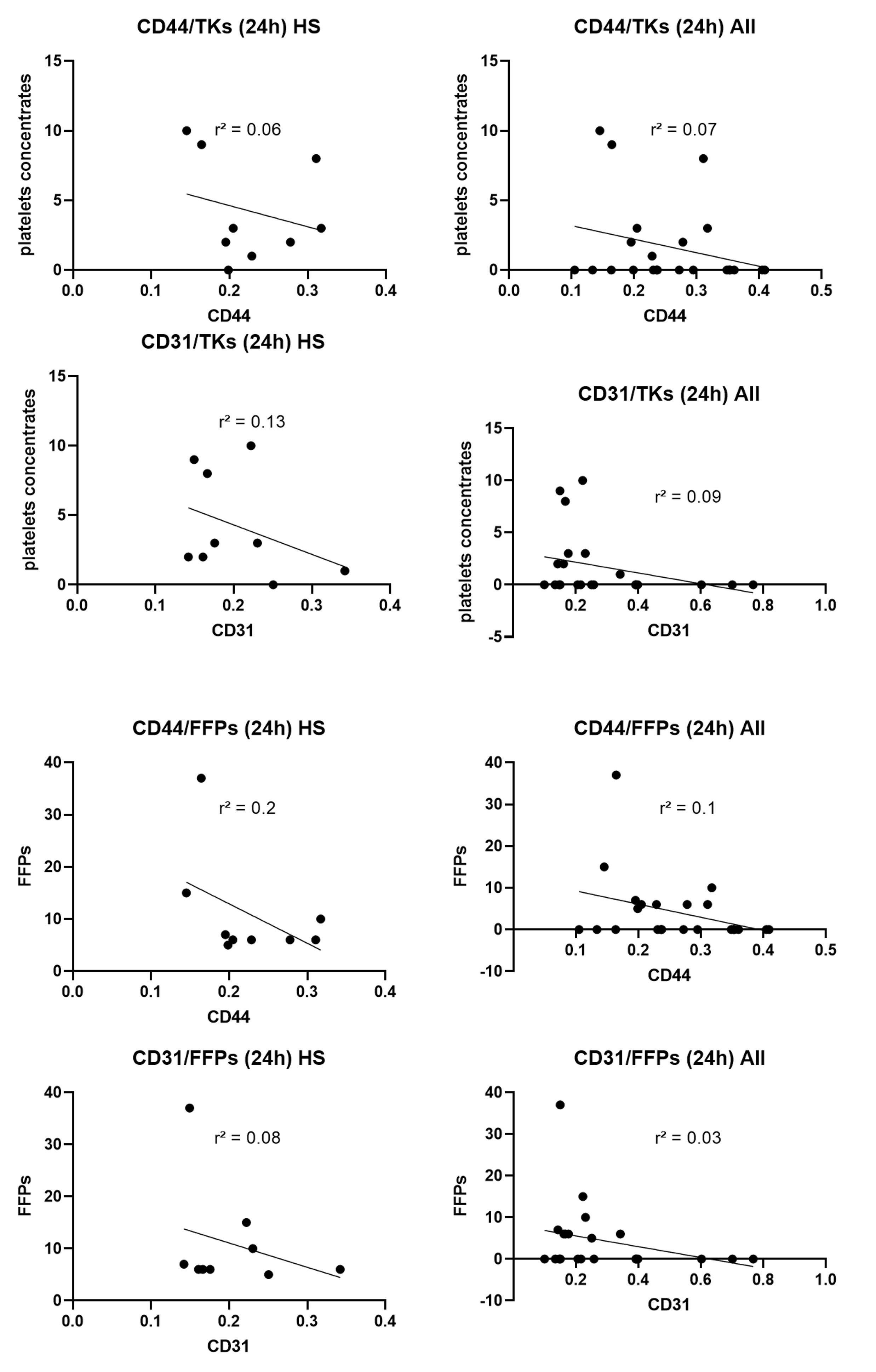

Supplement: Supplementary Figure 3 — Correlation Analysis of Fresh Frozen Plasma (FFPs) and platelet concentrates (TKs) and CD31/CD44+ EVs. All = both polytrauma groups, with and without hemorrhagic shock, HS = hemorrhagic shock [file Image_3.jpeg]
