# Supplementary material for: CD44+ and CD31+ extracellular vesicles (EVs) are significantly reduced in polytraumatized patients with hemorrhagic shock – evaluation of their diagnostic and prognostic potential
Source: Front Immunol. 2023 Aug 18;14:1196241. doi: 10.3389/fimmu.2023.1196241 (PMC10471799; doi:10.3389/fimmu.2023.1196241)
Supplement: Supplementary Table 1 — Correlation analysis of EV epitopes and lactate, hematocrit and crystalloids fluidics in HS group and in all patients. [file Table_1.docx]

|  | **HS patients (r^2^)** | | **All patients (r^2^)** | |
| --- | --- | --- | --- | --- |
|  | **CD31** | **CD44** | **CD31** | **CD44** |
| Lactate | 0.0017 | 0.013 | 0.045 | 0.002 |
| Lactate 24h | 0.005 | 0.09 | 0.09 | 0.15 |
| Crystalloids fluid | 0.03 | 0.01 | 0.02 | 0.003 |
| Hematocrit | 0.4 | 0.24 | 0.15 | 0.04 |
| Hematocrit 24h | 0.12 | 0.34 | 0.03 | 0.07 |
